# An oligo-swapping method: preparation of mismatch repair-monitoring substrate using a nicking endonuclease

**DOI:** 10.1016/j.mex.2025.103715

**Published:** 2025-11-07

**Authors:** Arato Takedachi, Mika Hayashida, Isao Kuraoka

**Affiliations:** Department of Chemistry, Faculty of Science, Fukuoka University, 8-19-1 Nanakuma, Jonan-ku, Fukuoka 814-0180, Japan

**Keywords:** Mismatch repair, Nicking endonuclease, *In vitro DNA construction*

## Abstract

Mismatch repair (MMR) contributes to accurate DNA replication by eliminating mismatched bases during DNA synthesis. Its importance is underscored by Lynch syndrome, a common hereditary colorectal cancer syndrome caused by MMR gene mutations. Therefore, quantification of MMR activity in human cells is important for diagnostic and therapeutic purposes.

To monitor MMR, we established a novel DNA plasmid, pBluescript II NLS-MC-EGFP-tdTomato (pBET2). After processing using our protocol called the oligo swapping method, the mismatch included in the pBET2 enables us to evaluate the MMR proficiency of living cells by detecting specific fluorescent markers. Since the method simply “swaps” short single-stranded DNA to create a single mismatch on a specific site of the plasmid, it is significantly easier and more user-friendly. Briefly, the nicking endonuclease Nt.*Bbv*CI produces a short single-stranded DNA region in the pBET2. Swapping is achieved by filling the region with a mismatched oligonucleotide, and T4 DNA ligase seals the gap. To isolate the mismatch substrate, the restriction enzyme *Spe*I-HF and T5 exonuclease clean up all contaminants, such as non-mismatch and/or non-covalently closed circular DNA substrates. Finally, the nicking endonuclease Nt.*Bsp*QI induces a nick on the final mismatch substrates that enhances the MMR reaction specifically on the target strand.

● This experimental method allows for the easy preparation of mismatched DNA substrates, in contrast to conventionally complex methods.

● This method enables the specific and efficient evaluation of MMR activity in living cells by using the mismatch substrate, a fluorescent marker.

● This method is applicable not only to MMR but also to the preparation of substrates for other DNA repair pathways, making it a valuable resource for both *in vitro* and *in vivo* experiments.

## Graphical abstract

The graphical abstract should summarize the contents of your article in a concise, pictorial form. Graphical abstracts should be submitted as a separate file. Image size: **please provide an image with a minimum of 531×1328 pixels (*h*×*w*) or proportionally more**. The image should be readable at a size of 5 × 13 cm using a regular screen resolution of 96 dpi. Preferred file types: TIFF, EPS, PDF or MS Office files.

**Figure:**
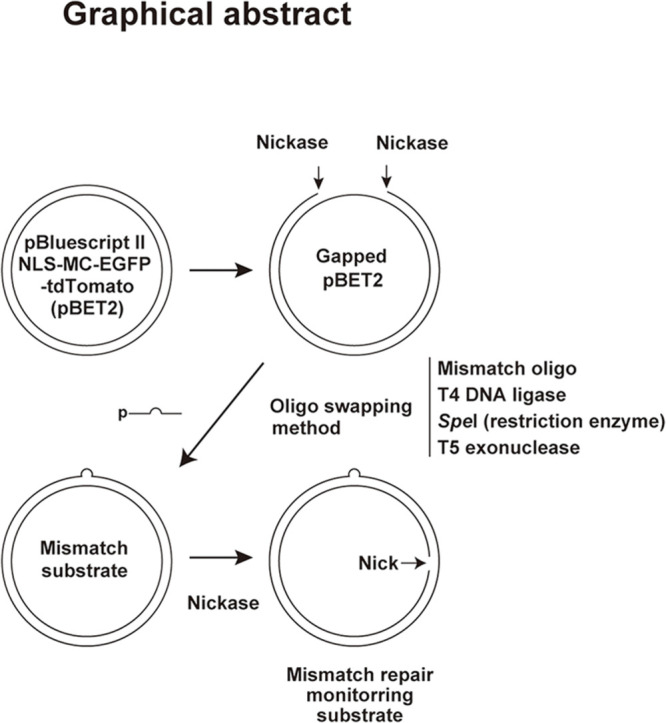

## Specifications table

This table provides general information on your method.

**Table:** 

**Subject area**	Biochemistry, Genetics and Molecular Biology
**More specific subject area**	DNA repair
**Name of your method**	Oligo-swapping methods
**Name and reference of original method**	Takedachi A, Matsuishi E, Mizusaki S, Nagasawa T, Fujikane R, Hidaka M, Iwai S, Kuraoka I. Novel plasmids for fluorescence-based evaluation of DNA mismatch repair in human cells. Mutat Res. 824 (2022):111,779. https://doi.org/10.1016/j.mrfmmm.2022.111779
**Resource availability**	Information on sequences and plasmids of pBluescript II NLS-MC-EGFP-tdTomato (pBET2) are available from Addgene (https://www.addgene.org). **Addgene ID** number is 240,411.

## Reagents/equipment

MATERIALS pBET2 (>500 ng/µL)

Oligonucleotides (FASMAC; reversed-phase chromatography grade). 5′-TCAGCGGTAC*C*AGTCACC-3′ containing a base pair mismatch (underline) that introduces A/C mismatch in pBET2 plasmid at the specific site.

Plasmid purification kit (Nanobond, Xtra Midi)

Nt.BbvCI: 10,000 units/mL (New England Biolabs [NEB]).

10x restriction endonuclease buffer (10X rCutSmartTM buffer: 500 mM potassium acetate, 200 mM Tris-acetate, 100 mM magnesium acetate, 1000 µg/mL recombinant albumin [pH 7.9 @ 25 °C])

25 mM ATP: 100 mM Adenosine 5′-Triphosphate (ATP) Disodium Solution (Fuji Wako) is diluted with H2O

T4 DNA ligase: 400 units/µL (Takara Bio)

SpeI-HF: 20,000 units/ml (NEB)

T5 exonuclease: 10,000 units/ml (NEB)

PCR purification kit (MACHEREY-NAGEL)

Agarose-ME classic type (Nacalai Tesque)

Ethidium Bromide Solution (EtBr; 10 mg/mL; Nacalai Tesque)

50x TAE buffer (40 mM Tris, 20 mM acetic acid, and 0.4 mM EDTA; Kanto Chemical)

Gel Loading Dye, Purple (6X) (NEB)

T4 polynucleotide kinase: 10,000 units/mL (Takara Bio)

Nt.BspQI: 10,000 units/mL (NEB)

2. LABORATORY EQUIPMENT

Thermoblock or thermal cycler

Microcentrifuge tubes (1.5 mL)

Centrifuge for 1.5 mL tubes

Electrophoresis power supply

Electrophoresis chamber

Spectrophotometer

Transilluminator

3.SOFTWARE

ImageJ: The amount of DNA substrate is estimated using ImageJ and an agarose gel.

## Background

Although replication polymerase is highly accurate, it can naturally insert incorrect bases during the synthesis of human genomic DNA. To overcome this, DNA polymerase possesses a notable "proofreading mechanism" that eliminates these errors and resynthesizes the DNA, thereby reducing the error rate to 1/10^7^ [1,2]. To further enhance this process and maintain genome stability, cells undergo a backup mechanism known as mismatch repair (MMR), which reduces the replication error rate to 1/10^9^. The MMR pathway is strand-specific, and the newly synthesized strand is removed to contain errors that result in mismatched bases during DNA synthesis [3].

In humans, MMR for removing errors, such as a single base pair mismatch, is believed to function in the following steps. Initially, the MutSα complex (a dimer composed of two distinct proteins, MSH2 and MSH6) interacts with the error-containing DNA and recognizes the error. Subsequently, it binds to MutLα (a dimer composed of two distinct proteins, MLH1 and PMS2) to form a complex. PMS2, a component of MutLα, generates a "nick" in the DNA in the proximity of the mismatched base. EXO1 exonuclease is then recruited to utilize this nick to degrade the error-containing DNA, and the DNA is then resynthesized. Finally, DNA ligase I joins the newly synthesized DNA, completing the repair process [[3], [4], [5]].

The vital role of MMR is highlighted in Lynch syndrome (LS), the most common hereditary colorectal cancer predisposition. LS is caused by inherited mutations in MMR genes (*MSH2, MSH6, MLH1*, and *PMS2*) that increase the risk of various cancers, including colorectal, endometrial, and ovarian cancers. Therefore, MMR research is crucial for the diagnosis, prevention, and treatment of LS [6,7].

Current MMR deficiency tests such as microsatellite instability testing and protein immunostaining are indirect methods for evaluating MMR proficiency. Although *in vitro* MMR activity assays can directly assess the entire process, they require specialized technical skills [8,9].

Preparation of DNA substrates, especially those with mismatches, is a laborious task.

**Fig. 1:**
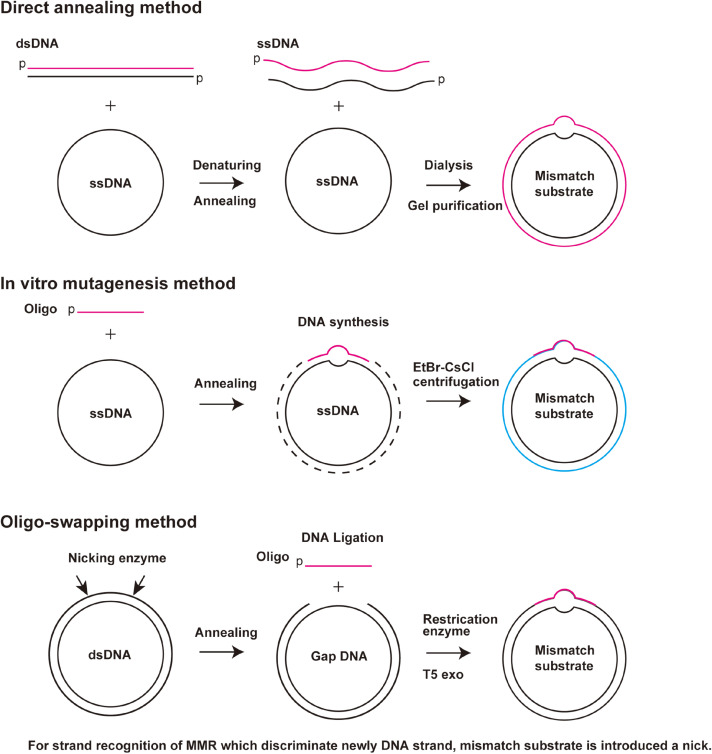
A) Direct annealing method. Mismatch substrates are prepared by annealing linearized double‐stranded plasmid DNA (*e.g.* replicative form of M13 phage or phagemid) and single‐stranded circular DNA (*e.g.* M13 circular ssDNA or phagemid ssDNA) in the presence of high formamide concentration. This reaction mixture is dialyzed while dropping from a high to progressively lower formamide concentrations. Nicked circular molecules are purified via agarose gel electrophoresis and electroelution. B) *In vitro* mutagenesis method. Mismatch substrates are prepared by annealing synthetic oligonucleotides and single‐stranded circular DNA. After annealing, DNA polymerase and DNA ligase synthesize and seal the new DNA strand, resulting in a circular DNA containing the single mismatch. Closed circular DNA is isolated via CsCl/EtBr density gradient centrifugation. C) Oligo-swapping method. Nicking endonuclease generates a region of ssDNA in plasmid. Annealing with synthetic oligonucleotides, DNA ligase seals it to produce closed circular DNA. Treatment with restriction enzyme and T5 exonuclease isolates the closed circular DNA containing the mismatch.

Three main methods can be used to produce DNA substrates with mismatches (Fig. 1).

1. Direct annealing method: Annealing of single-stranded (ssDNA) and double-stranded (dsDNA) DNA, followed by purification of the plasmid containing the mismatch [10,11].
2. *In vitro* mutagenesis method: Annealing of ssDNA and a synthetic mismatch oligonucleotide, followed by *in vitro* DNA synthesis and ligation to create the mismatched substrate [12,13].
3. Oligo-swapping method: Nicking endonuclease recognition sites are used to create a gap in the plasmid, into which a synthetic oligonucleotide is ligated to introduce a mismatch [14,15].
4. The direct annealing and *in vitro* mutagenesis methods are complex and costly, because they involve the use of phage ssDNA and dsDNA and/or *in vitro* DNA synthesis.

We have previously developed a living cell assay using the pBSII EGFP-tdTomato reporter plasmid [16]. This assay detects mismatch correction by restoring green fluorescent protein (GFP) expression, with tdTomato indicating the transfection efficiency. We improved this assay by modifying the plasmid (pBSII EGFP-tdTomato reporter plasmid ver.2 [pBET2]) to include a nickase site for introducing a synthetic mismatch oligonucleotide [15]. This modified plasmid efficiently measure MMR repair capacity in living cells.

## Method details

**Fig. 2:**
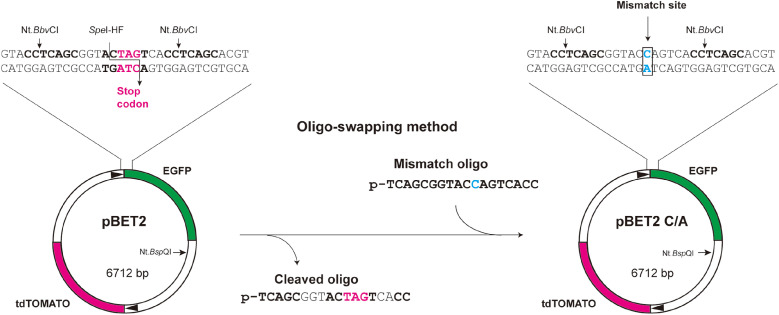
Schematic diagram of the mismatch substrate pBET2 C/A synthesized from the reporter plasmid pBET2 by the oligo-swapping method. The plasmid DNA, pBET2 (for sequence, see Addgene ID 240,411), was designed with two nicking sites for the endonuclease Nt.*Bbv*CI to enable the oligo-swapping method. Using this method, the mismatched substrate pBET2 C/A is synthesized from pBET2. Successful repair of the C/A mismatch via MMR in living cells results in the detection of GFP protein within the nucleus. The co-expressed dtTOMATO protein is used as a control to confirm successful transfection.

The plasmid DNA, pBET2, was designed with two nicking sites for the endonuclease Nt.*Bbv*CI allows the oligo-swapping method and assessment of MMR proficiency in living cells by detecting GFP. Furthermore, it contains an Nt.*Bsp*QI nicking site, a recognizable marker that supports the MMR repair machinery in distinguishing the target strand for repair and a nuclear localization signal (NLS) that facilitates GFP expression in the nucleus. Using the oligo-swapping method, the mismatched substrate pBET2 C/A is synthesized from pBET2 (Fig. 2).

1. To prepare the pBET2 plasmid, purify using a conventional plasmid purification kit (Nanobond, Xtra Midi). The typical total yield of DNA from this kit is approximately 500 µg, which is standard for a Midi-prep kit. The DNA is eluted at a high concentration and subsequently adjusted to a final concentration of 0.5 mg/mL. A DNA concentration of at least 500 ng/uL is recommended. Of the DNA solution, 0.4 µL is kept as control for an agarose gel loading sample. Store the sample at 20 °C prior to gel loading (Fig. 3A, lane1:200 ng/lane).

**Fig. 3:**
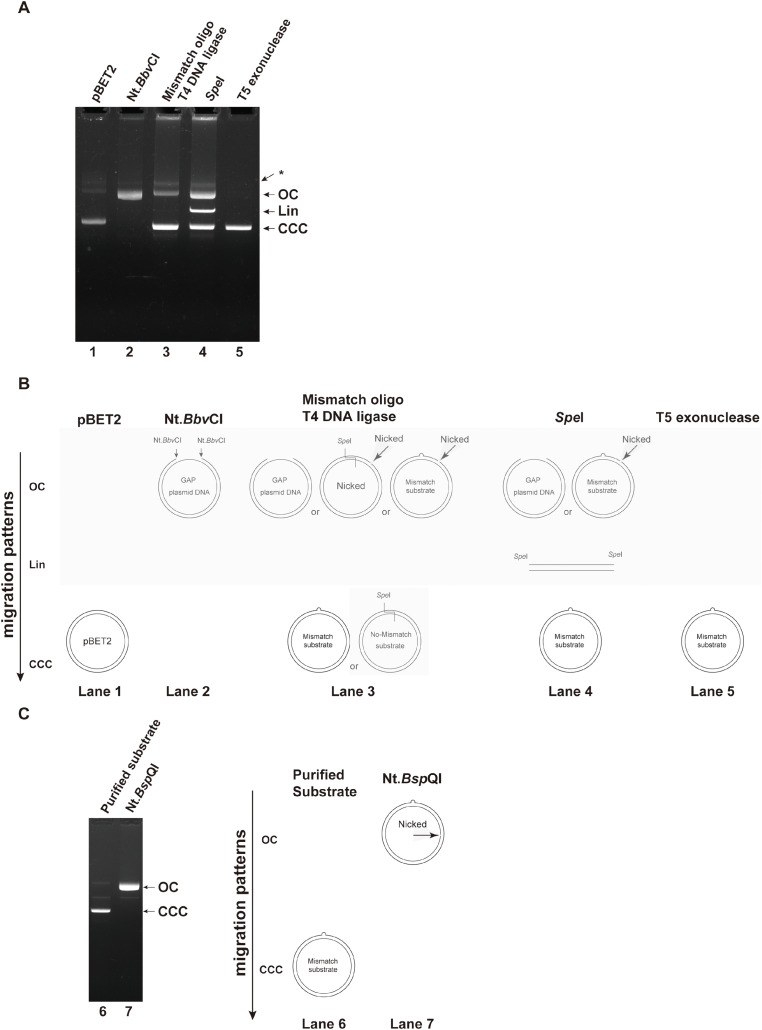
Aliquots from various steps of the purification were analyzed on 0.8 % agarose gel, and the DNA substrates were visualized via EtBr staining. A) Lane 1, pBET2 (Method details, step 1); lane 2, Nt.*Bbv*CI-treatment (Method details, step 2); lane 3, T4 DNA ligase-treatment (Method details, step 4); lane 4, *Spe*I-HF- treatment (Method details, step 5); and lane 5, T5 exonuclease-treatment (Method details, step 6). Open circular DNA (OC), linear DNA (Lin), and covalently closed circular DNA (CCC) are indicated by arrows. B) DNA conformation in each lane. In lane 1, the purified pBET2 plasmid is a closed circular DNA. In lane 2, gapped pBET2 is an open circular DNA. In lane 3, gapped and non-reacted DNA are open circular DNA, while mismatch and non-mismatch DNA are closed circular DNA. In lane 4, nicked DNA and non-mismatch DNA are digested with *Spe*I-HF, resulting in linear DNA. In lane 5, the subsequent step entails the removal of linear and gapped DNA by T5 exonuclease to isolate mismatch DNA, pBET2 C/A. A single nick site for MMR s introduced. In lane 6, pBET2 C/A is purified using a standard PCR purification kit. In lane 7, the DNA is digested by a nicking endonuclease, forming open circular DNA.
2. To make the gap region of ssDNA on the plasmid, make a mixture containing 80 µL (40 µg: approximately 10 pmol) of pBET2 (500 ng/µL), 4 µL of Nt.BbvCI (10,000 units/mL), 20 µL of 10x restriction endonuclease buffer, and 96 µL of H_2_O. Incubate the mixture at 37 °C for 2 h. Stop the reaction at 80 °C for 20 min. Keep 1 µL of the reaction mixture as an agarose gel loading sample to ensure that gapped DNA is produced by Nt.*Bbv*CI (Fig. 3A, lane 2). Store the sample at - 20 °C until loading onto 0.8 % agarose gel.
3. For the annealing reaction, add 1 µL of mismatch oligonucleotide (200 pmol/µL), 25 µL of 10x restriction endonuclease buffer, and 24 µL of H_2_O. Incubate the mixture at 95 °C for 5 min, 70 °C for 5 min, and then at room temperature for 15 min.
4. For the ligation reaction (sealing of the oligonucleotide into the gapped DNA), add 10.8 µL of 25 mM ATP, 7 µL of T4 DNA ligase (400 units/µL), and 2.5 µL of H_2_O. Incubate the mixture at 16 °C for overnight. Stop the reaction at 65 °C for 10 min. Keep 2.7 µL of the reaction mixture as an agarose gel loading sample to ensure that the gapped DNA is sealed with annealed mismatch oligonucleotide by T4 DNA ligase (Fig. 3A, lane 3). Store the sample at - 20 °C until loading onto 0.8 % agarose gel.
5. For the restriction endonuclease reaction to remove non-mismatch DNA, add 2 µL of *Spe*I-HF (20 units/µL). Incubate the mixture at 37 °C for 1 h. This restriction enzyme cleaves the remainder of the plasmid which does not contain the mismatched oligonucleotide. Stop the reaction at 80 °C for 20 min. Keep 2.7 µL of the reaction mixture as an agarose gel loading sample to check that the newly synthesized DNA containing the mismatch oligonucleotide is undigested with *Spe*I-HF (Fig. 3A, lane 4). Store the sample at - 20 °C until loading onto 0.8 % agarose gel.
6. For the T5 exonuclease treatment to remove linear and open circular DNA, add 4 µL of T5 exonuclease (10 units/µL). Incubate the mixture at 37 °C for 1 h. Keep 2.7 µL of the reaction mixture as an agarose gel loading sample to check that T5 exonuclease isolates covalently closed circular DNA (Fig. 3A, lane 5). Store the sample pBET2 C/A at - 20 °C until loading onto 0.8 % agarose gel.
7. Analyze the reaction products using a 0.8 % agarose gel containing EtBr (Fig. 3). All samples (200 ng/lane) containing the gel-loading dye are loaded onto the gel. The starting material, the pBET2 DNA plasmid, contains both closed-circular (∼90 %) and open-circular (∼10 %) DNA in lane 1 (Fig. 3A and 3B). After Nt.*Bbv*CI treatment, the sample showed open-circular DNA, indicating that it was the gap region in pBET2 in lane 2 (Fig. 3A and 3B). T4 DNA ligase sealed the gapped DNA with an annealed mismatch oligonucleotide (lane 3). The newly sealed DNA is covalently closed, resulting in a circular DNA duplex containing either a single or no mismatch, and unsealed products are observed as open-circular DNA (Fig. 3A and 3B, lane 3). *Spe*I-HF digests non-mismatched substrates, resulting in closed-circular to linear DNA (Fig. 3A and 3B, lane 4). The T5 exonuclease (T5 exo) cleaves linear or nicked open-circular DNA. Thus, the final DNA products (Fig. 3A and 3B, lane 5) and the mismatched substrates (pBET2 C/A) are isolated. It was estimated to be approximately 95 % pure on an agarose gel using the ImageJ software (Fig. 3A, lane 5).
8. Use a PCR purification kit (MACHEREY-NAGEL) to purify the mismatched DNA substrate from the reaction mixtures (Fig. 3C, lanes 6, 200 ng/lane). EtBr-CsCl gradient centrifugation and/or gel purification methods are also available. Quantify the final DNA solution using a spectrophotometer. The DNA concentration is adjusted to approximately 100 ng/µL. Purified DNA may be stored in aliquots at −20 °C for a few months.
  Option: (This step is performed after purifying pBET2 C/A.)
9. For strand recognition of MMR, which discriminates the newly synthesized DNA strand, prepare a mixture containing 50 µL of the purified DNA mismatch substrate (100 ng/µL), 5 µL of Nt.*Bsp*QI, 10 µL of 10x restriction endonuclease buffer, and 35 µL of H_2_O. Incubate the mixture at 50 °C for 2 h (Fig. 3C, lane 7; 200 ng/lane). Stop the reaction at 80 °C for 20 min. At this step, the DNA concentration is 50 ng/µL (5 µg).

## Method validation

The pBET2 plasmid contains a gene encoding a green fluorescent protein (EGFP) with an inactivating mismatch. Correcting this mismatch via MMR restores EGFP gene expression. It also harbors a gene that encodes a red fluorescent protein (dTomato), which indicates transfection efficiency. Therefore, EGFP is detected when mismatched substrates (pBET2 C/A) are introduced into normal MMR-proficient cells (*e.g.*, HeLa and U2OS) but not in MMR-deficient cells (*e.g.*, DLD1, LoVo, and HCT116).

The presence of an A:C mismatch in the DNA substrate can be confirmed by DNA sequencing, which can performed on each purified plasmid DNA strand [14].

## Limitations

None

## Ethics statements

None

## CRediT authorship contribution statement

**Arato Takedachi:** Conceptualization, Writing – review & editing. **Mika Hayashida:** Data curation, Investigation, Validation. **Isao Kuraoka:** Supervision, Conceptualization, Writing – review & editing, Funding acquisition.

## Declaration of competing interest

The authors declare that they have no known competing financial interests or personal relationships that could have appeared to influence the work reported in this paper.

## Data Availability

The authors do not have permission to share data.
